# Intestinal Strictures Post-Necrotising Enterocolitis: Clinical Profile and Risk Factors

**Published:** 2014-10-20

**Authors:** Nilkant Phad, Amit Trivedi, David Todd, Anil Lakkundi

**Affiliations:** 1 Grace Centre for Newborn Care, Children’s Hospital at Westmead, Westmead, NSW, Australia; 2 The Canberra Hospital, Garran, ACT, Australia; 3 John Hunter Children’s Hospital, New Lambton Heights, NSW, Australia

**Keywords:** Necrotising enterocolitis, Intestinal strictures

## Abstract

Background: Intestinal stricture is an important complication of necrotising enterocolitis (NEC). We aimed to describe clinical profile and identify the risk factors for post-NEC intestinal strictures.

Method: A retrospective study of infants with NEC over 10 year period.

Results: Of the 61 infants with NEC, 18 (29.5%) developed intestinal strictures. Leucocytosis and longer length of bowel resection during acute stage of NEC was associated with a later diagnosis of intestinal stricture. Infants with NEC who did not develop stricture had non-specific intestinal dilatation on abdominal x-ray during acute NEC. Intestinal strictures were diagnosed at a median interval of 34 days after NEC. Majority of strictures (67%) occurred in the colon. A significant proportion (77%) of infants with intestinal stricture had associated co-morbidities. No mortality occurred in infants with intestinal strictures.

Conclusion: The incidence of post-NEC intestinal stricture is high but development of stricture is difficult to predict. Leucocytosis during NEC and length of bowel resected at surgery may be associated with development of post-NEC intestinal stricture. A substantial number of infants with post-NEC intestinal stricture fail to thrive, have co-morbidities and need prolonged hospitalisation.

## INTRODUCTION

Neonatal necrotising enterocolitis (NEC) is one of the most common surgical emergencies in neonatal intensive care units (NICU) with a reported incidence of 2.6% to 6.6%, depending on the gestational age of infant at birth [1,2]. Most cases of NEC are managed conservatively and surgical intervention is required in infants with intestinal perforation or deteriorating clinical or laboratory parameters despite optimum medical treatment [3,4]. With improved survival of many seriously ill infants with NEC, a substantial number of infants present with intermediate or long-term sequelae [5]. Intestinal stricture following NEC is a well-known complication of the healing process that follows ischaemic injury to the inner muscular layer of the intestine, during the acute episode [6]. Intestinal strictures develop irrespective of the mode of management of the acute episode of NEC and tend to have a strong preference for the left colon (splenic flexure, descending and sigmoid colon) [3]. Strictures manifest at a variable period after the acute NEC. Several studies have described significant mortality and morbidity secondary to intestinal strictures [3,5,7]. However, there is paucity of recent literature describing the natural history of intestinal strictures following NEC [8]. 

The aim of this study was to describe clinical profile, complications and outcomes of post-NEC intestinal strictures in a surgical neonatal unit over a period of 10 years and to identify potential risk factors during the acute NEC episode that could predict development of post-NEC intestinal stricture.

## MATERIALS AND METHODS

This was a retrospective study of infants who were treated for NEC in a neonatal unit of a children’s hospital from 1st January 2000 to 31st December 2010. All infants admitted to the neonatal unit during the study period with a diagnosis of NEC were eligible for inclusion. The study population was identified from the hospital’s electronic database by quizzing the database with search terms “Necrotising Enterocolitis”, “NEC” “Stricture” and “Intestinal Stenosis”. The infants with congenital anomalies and infants who died during acute NEC were not included in the study. 

The electronic medical records and discharge summaries were reviewed. Demographic information, clinical indicators of severity of NEC such as haematological investigations, findings on abdominal x-ray, mode of management of the acute episode of NEC, length of bowel resected at surgery and histopathology of resected bowel segment were collected. All the abdominal X-rays were reported by a paediatric radiologist. The infants were classified into Stage I, Stage II or Stage III NEC using the modified Bell’s criteria. 

For the infants who had post-NEC strictures, age at the diagnosis of stricture, time interval since diagnosis of acute NEC, weight at diagnosis of stricture, signs at presentation, results of intestinal contrast study, number and site of strictures, complications attributable to strictures, mode of management of the stricture, time required to attain full enteral feeds and the length of hospital stay were recorded. All Infants with NEC were followed up in the hospital’s outpatient clinic until at least 3 months of corrected age. 

Ethical approval was granted by the local Human Research Ethics Committee. After the database was accessed and the medical records examined, de-identified study data were recorded electronically and password protected by the primary investigator.

Statistical Analysis:

Data was analysed using SPSS software (IBM SPSS statistics for window, release 20.0.0) Chicago, IL, USA. Chi Square test, Fisher’s Exact test and Mann Whitney test were used as appropriate. A p-value of less than 0.05 was considered statistically significant.

## RESULTS

We identified 72 infants with NEC during the study period. 11 infants died due to NEC and were excluded. 61 infants (28 male and 33 female) with NEC were included in the study. The baseline characteristics of infants with or without strictures were similar (Table 1). Modified Bell’s stage I NEC occurred in 15/61 (24.5%) infants, Stage II in 22/61 (36%) and Stage III in 24/61 (39.5%) infants. Surgical intervention was required in 28/61 (46%) infants and 33/61 infants (54%) were managed conservatively. Intestinal strictures developed in 18/61 (29.5%) infants. The incidence of stricture was 35.5% (10/28) in the surgically managed subgroup and 24% (8/33) in the conservatively treated subgroup. 

**Figure:**
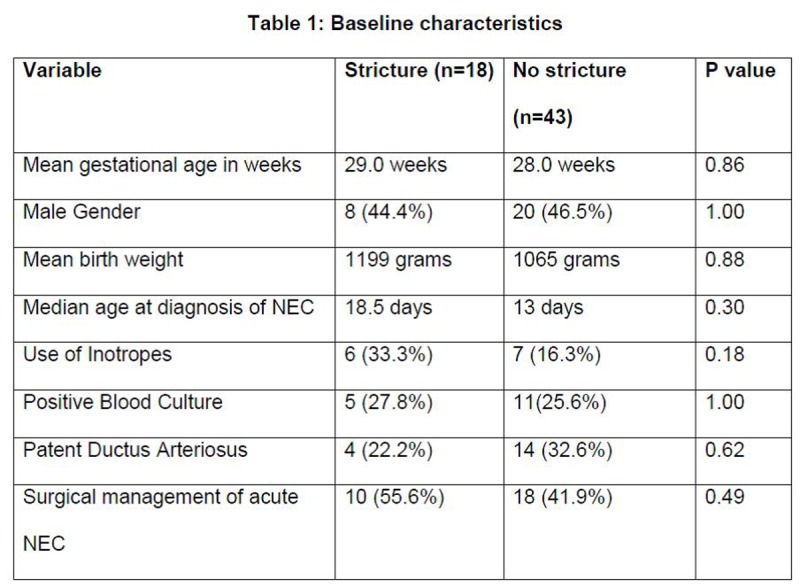
Table 1: Baseline characteristics

The infants who developed intestinal strictures were more likely to have an elevated white cell count (> 25000/cmm) at the time of acute episode of NEC (p = 0.01) (Table 2). Infants with Bell’s stage I disease and non-specific intestinal dilation on x-ray were less likely to develop a stricture (p = 0.05 and 0.02 respectively) (Table 3). Of the intestinal perforations during acute NEC (17/61 infants) caecal perforation posed a borderline risk for development of strictures (P=0.05). In infants that required surgical intervention for NEC, longer length of resected bowel was significantly associated with development of strictures (p=0.02) (Table 3).

**Figure:**
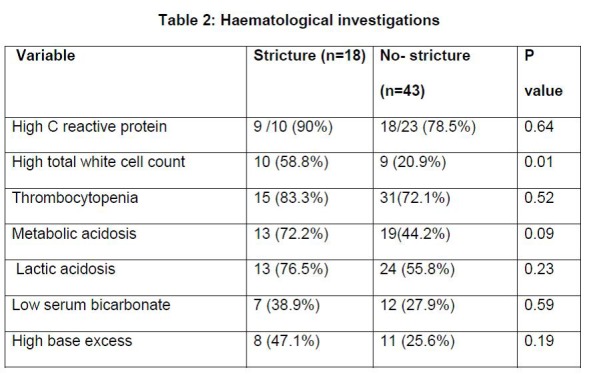
Table 2: Haematological investigations

**Figure:**
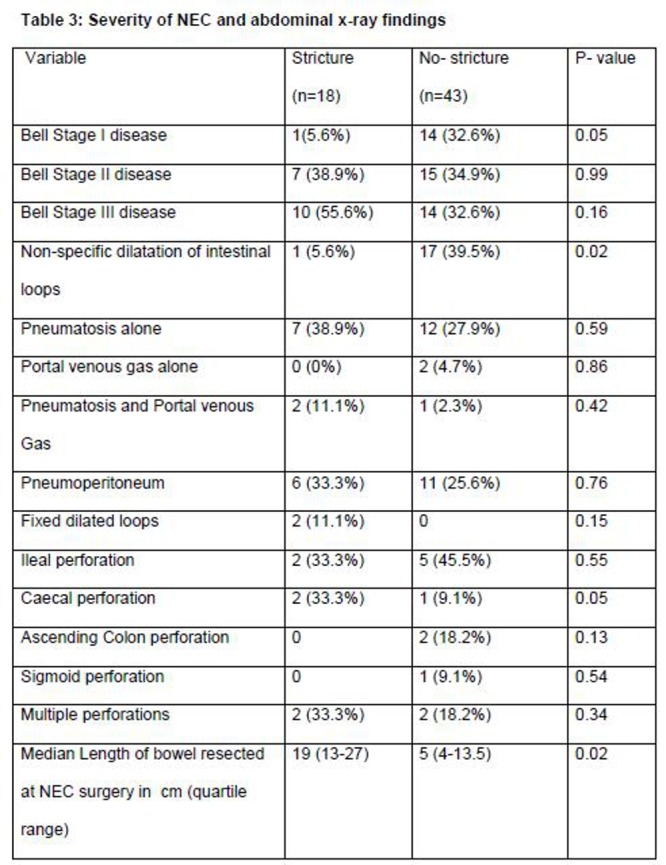
Table 3: Severity of NEC and abdominal x-ray findings

In our series of 18 infants with intestinal strictures, the median time interval between NEC and diagnosis of the intestinal stricture was 34 days (range 27.5-62 days). In 7/18 (39%) infants the strictures were diagnosed by the routine contrast study prior to reversal of stomas to re-establish gastro-intestinal continuity. The remaining 11 infants had symptoms of intestinal obstruction and the diagnosis of intestinal strictures was established on a contrast study.

In 12/18 (67%) infants the strictures were located in the colon and in 6/18 (33%) the site was small bowel. Amongst the isolated colonic strictures, four were located in the transverse and descending colon each and one in the ascending colon. Three infants had strictures involving more than one segment of colon. In six infants with stricture of the small bowel, five were located in the terminal ileum and one in the proximal ileum. All small bowel strictures were isolated. 

The complications noted in infants with intestinal strictures were intestinal perforation (2/18) bacterial sepsis (2/18), severe electrolyte imbalance in (4/18), hypoalbuminemia requiring albumin transfusion (8/18) and significant cholestasis in (9/18). Seven infants with intestinal stricture had growth failure with weight that dropped two major centile lines to well below 10th centile between the diagnosis of acute NEC and the diagnosis of stricture. Median time required to attain full enteral feeds after repair of stricture was 25 days (range 14-67.7 days) and the median duration of hospitalisation for stricture and related issues was 33 days (range 21-81days). No deaths occurred in the infants with strictures in our cohort.

## DISCUSSION

Intestinal strictures following NEC in neonates were first reported by Rabinowitz et al in 1968.[9] There is paucity of studies looking at risk factors for post-NEC intestinal strictures [8]. With improved perinatal care in the last 20 years we sought to evaluate the pattern of post-NEC intestinal strictures. Our study had a sizeable number (18) of infants with intestinal stricture in comparison to the previous studies [5-11]. 

Our findings of the incidence of stricture, the median time to diagnosis of stricture since the acute episode of NEC, location of strictures and lack of significant association between development of post-NEC stricture and mode of management of acute NEC were similar of the previous reports [5,7,11-15]. 

The intestinal strictures result from collagen deposition, fibrosis and wound contraction over a variable period following an episode of acute inflammation. We examined various markers of inflammation and severity of illness at the time of acute NEC. We observed significant association between presence of high white cell count during acute NEC and length of bowel resected at NEC surgery and the occurrence of intestinal strictures. These findings have not been described in the literature previously. They could be of prognostic significance and are biologically plausible. We hypothesize that presence of large number of white cells resulting in heightened immune mediator response locally may precede development of stricture and more length resection to be marker of more extensive disease . Contrastingly, level of C- reactive protein (CRP) that has been reported as a part of a scoring system to predict development of stricture did not attain prognostic relevance in our study.[16] Thrombocytopenia and metabolic acidosis at the time of acute NEW were not predictors of later strictures. 

Growth failure and life threatening complications of strictures such as intestinal perforation and sepsis are well described in literature [3,5]. We found severe electrolyte abnormalities, hypoalbuminemia, significant derangement of liver functions as associated morbidities in infants with intestinal stricture. Our observation of morbidity associated with intestinal stricture (in more than three fourths of infants) was higher than that reported by Janik et al (30%) [7]. This difference may be due to more variables studied in our study population and comparatively larger infants in the report by Janik et al. Mortality ranging from 8-25%, secondary to intestinal stricture related problems such as intestinal perforation, aspiration pneumonia and sepsis in the peri-operative period, has been reported in the literature [3’7]. We did not observe any mortality in infants with strictures. This difference could be due to improved neonatal care in the last two decades.

This is a retrospective study from single neonatal unit with a relatively small sample size and might not be powered to detect small differences between the study groups. Infants included in this study were referred from other neonatal units for surgical evaluation and could represent the severe end of NEC spectrum making the observations not applicable to perinatal settings that medically manage infants with NEC.

## Conclusion

Our study offers a recent insight into the impact of improved care on the clinical profile and outcomes of post-NEC strictures. The overall incidence of intestinal strictures has remained high and similar irrespective of the mode of management of NEC. Prediction of development of intestinal stricture following NEC is difficult. However, leucocytosis during acute NEC and the length of bowel resected at surgery could be potential predictors of post-NEC stricture. A substantial number of infants with strictures failed to thrive; the majority had morbidity and required prolonged hospitalisation. 

Implications for current Practice:

All infants with confirmed NEC should be observed closely and investigated for stricture in the presence of any symptom of persistent gastrointestinal disturbance irrespective of initial mode of management. Physical growth, liver functions and serum electrolyte levels of infants with post NEC strictures should be closely monitored and nutrition proactively managed.

## Footnotes

**Source of Support:** None

**Conflict of Interest:** None

